# Psychometric Properties and a Preliminary Validation Study of the Italian Brief Version of the Communication Styles Inventory (CSI-B/I)

**DOI:** 10.3389/fpsyg.2020.01421

**Published:** 2020-06-22

**Authors:** Pierluigi Diotaiuti, Giuseppe Valente, Stefania Mancone, Angela Grambone

**Affiliations:** Department of Human Sciences, Society and Health, University of Cassino and Southern Lazio, Cassino, Italy

**Keywords:** communication styles, confirmatory analysis, impression manipulativeness, emotionality, expressiveness

## Abstract

People will typically develop a communication style that tends to be coherent with their own fundamental personality traits. The current debate on communication style acknowledges the construct of adaptive behavior as an appropriate area where to include both the strictly personal aspects and social learning and cultural assimilation, which translate into communicative style as a specific form of adaptation integrating the behavioral and personality perspectives. Due to the lack of instruments in the Italian psychometric scenario to assess communication styles, the present study included the translation and validation of the Italian short version of the *Communication Styles Inventory* (CSI-B/I). Methods. The CSI-B/I was administered to a sample of 1,044 participants, while the concurrent validity was tested through a second administration to 518 participants along with the MPP (*Multidimensional Personality Profile*). Results. Confirmatory factor analysis bore out a three-factor solution (including 18 items) with good indices of adaptation to data, e.g., χ2/df = 1.251, RMSEA = 0.027, RMSEA 90% CI = 0.008–0.040, GFI = 0.958, AGFI = 0.937, CFI = 0.983 and NFI = 0.922. The CSI-B/I allows to measure three main dimensions of the communication style: impression manipulativeness; emotionality; expressiveness. Internal consistency reliability and significant correlations with the MPP supported the concurrent validity of the tool. Conclusion. By virtue of its good psychometric properties, CSI-B/I represents an important addition to the assessment in multiple contexts: companies, institutions, staff selection, individual and group profile analysis, coaching, psychotherapy, counseling, career guidance.

## Introduction

In the field of communication studies the problem with identifying interactive communication styles has been dealt with on the one hand as an identification of a stable and recurring model of communication practices and on the other hand as an interpretation of the specific influence of individual personality characteristics on the verbal and non-verbal manifestations of people. Currently, the two approaches seem to be usefully converging toward an integrated solution of these two perspectives (Bakker-Pieper and De Vries, 2013; De Vries et al., 2013).

In defining communication style, researchers have for the most part used a description and a classification of the relational messages that individuals share in an interaction. Initially, Norton (1978, 1983) defined communication style as a relatively stable model of verbal and non-verbal interaction, associated to specific expectations of the role of the person. Besides identifying nine different styles of communication^1^, he also added another construct referred to as *communicator image*, which measures the individual’s rating of how he/she perceives his/her own efficacy in communication. Various researchers have subsequently criticized the low level of internal reliability of Norton’s scale (Rubin and Martin, 1994; Horvath, 1998).

Hansford and Hattie (1987) while proceeding on the basis of Norton’s work, have instead conducted a factorial structure analysis and identified five dimensions of communicative style (dominant, expressive, relaxed, animated, attentive) and they have isolated two second-order factors on the scale: *animated-dominant* and *supportive-attentive.*

Other researchers from an organizational ambit (Bolton and Bolton, 1984) have, however, developed a more social model of communication styles, based on two fundamental dimensions of social behavior: *assertion* and *reactivity.* The first aspect measures how much dominance and self-confidence is manifested by the person during communication; the second expresses the person’s skills in conveying emotional reactions through communication. Bolton & Bolton also added another social dimension for *versatility*, that is, the individual’s capability of changing his/her style whenever he/she realizes that the situation is putting him/her under pressure. Recent contributions enhance the appropriateness of the communicative style for the achievement of the organizational goals (Crews et al., 2019; Yang et al., 2020).

Other studies on communication styles have considered the effect of the cultural component. Consequently, a distinction has been made between modes of communication that are little influenced by the cultural context of belonging and modes of communication that are strongly influenced and dependent on the cultural context of reference. In the first case individuals tend to send explicit and direct messages, while in the second case the messages are indirect and contextualized and always need to be adequately interpreted in order to avoid misunderstanding (Croucher et al., 2012).

Kapoor et al. (2003) in their research have shown that the first style of communication is typical of individualistic cultures, while the second is more frequently found in cultures similar to the collective model. The research of Gudykunst et al. (1996) was also oriented toward the cultural-comparative ambit and produced the *Scale of Communication Styles (CSS)* made up of eight main dimensions (ambiguous and indirect communication, interpersonal sensitivity, positive perception of silence – more frequent in cultures that are highly dependent on their context; inference of significance, use of emotion, dramatization, authenticity, precision – mainly associated with individualistic cultures). Successively, Leung and Bond (2001) in their analyses, extracted three second-order factors from these eight dimensions: verbal involvement, attention toward others, management of emotions and silence.

The recent contribution of Reinout De Vries et al. (2009) stands out among these studies on communication styles. He used a lexical type approach to describe the communicative behavior. First he looked for the underlying factors in an ample group of adjectives and verbs belonging to the communicative sphere. From this he extrapolated seven factors: precision, reflexion, expression, support, emotionality, kindness, threatening.

The development of the work of De Vries and his collaborators resulted in the construction of the *Communication Styles Inventory (CSI)*, which presently constitutes, together with the *Scale of Communication Styles (CSS)* by Gudykunst, one of the few instruments available for the *general measurement* of communication styles adopted by an individual. However, in the literature we can find various instruments for the measurement of communication styles for specific ambits such as schools (Harkin et al., 1999), medical communication (Ong et al., 1995), leadership (Bechler and Johnson, 1995), and marketing (Dion and Notarantonio, 1992).

Beatty et al. (1998) is notable for having investigated the relationship between communicative behavior and personality traits. In this sense the communication style is considered to be the disposition of the person toward a certain kind of communication. Strictly speaking, communication styles are not personality traits but they emerge under the influence of these traits and the environment. Behavioral dispositions tend to generate stable relationships between the situations and the reactions of a person (Asendorpf, 2004).

According to Schulz von Thun (2003) these styles constitute a specific mode of contact, communication and management of relationships with other people. They are associated with need “flows,” states of mind and intentions that manifest their force through words and non-verbal signals. The various styles do not exclude each other but rather they come together in the person in a specific combination which as a whole expresses the individual’s preferred model of contact. The five-factor model of personality is currently a reference point for identifying the fundamental tendencies specific to each person, stable over time and mostly hereditary: neuroticism, extroversion, openness to experience, friendliness and conscientiousness (McCrae et al., 2000).

Taking this model into consideration in order to apply it to a communication theory means interpreting the communication style of an individual as a form of specific adaptation, relatively stable, of his/her behavioral models under the influence of his/her personality. This means that a person will develop a communication style that tends to be coherent with his/her fundamental personality traits. For example, a person with a prominent friendly trait will prefer to express himself in a kind and polite way rather than use aggressive expressions.

Cole and McCroskey (2000) examined the relationships between two personality models and the communication styles: the first model was the model with three factors (neuroticism, extroversion, psychosis) while the second was the now classic McCrae and John (1992). The results showed that half of the variances of the scores on the communication measurement scales were explained by the *assertive* dimension, while two thirds of the variances were due to the *reactive* dimension. At the moment these are the two main dimensions extrapolated and confirmed by diverse research on communication styles. Cole and McCroskey (2000) concluded their study by affirming that the communication style could be foreseen very clearly in the characteristics and personality traits of the individuals.

The limit of a model that refers to only two dimensions is without doubt that of an excessive simplification of reality. To contain this risk and at the same time safeguard the principles of precision and parsimony, other researchers have developed a model, defined as the “interpersonal circumplex” model, where the different modes of interaction are collocated in a circular area divided into eight sectors. The circular area is also defined by two main orthogonal, and therefore independent, dimensions (dominance and love). The sectors, each describing an interpersonal mode of behavior, are particular combinations of the two main dimensions: confident-arrogant; gregarious-extrovert; warm-kind; modest-naive; insecure-submissive; detached-introvert; cold-indifferent; arrogant-calculating. The adjacent sectors correlate positively with their adjacent sectors and negatively with their opposites; whereas there are no significant correlations with the orthogonal sectors (Leary, 1957; Wiggins, 1979; Wiggins et al., 1988, 1989).

There is general agreement that empirical research could find evidence of good collocations of communication styles inside an interpersonal circumplex model (Waldherr and Muck, 2011). However, although there has been some preliminary research orientated in this sense, to date there has not yet been a completely exhaustive study on this project (Sorenson and Savage, 1989; Leung and Bond, 2001; Muck, 2003; Riggio and Carney, 2003).

The current debate on the definition of communication style acknowledges the construct of adaptive behavior as an appropriate area where to include both the strictly personological aspects, and the social learning and cultural assimilation aspects that inevitably influence the communicative mode. The effects relating to the type of education received and the culture and environment in which the individual lives can significantly overlap with the personality trait (Liliweri, 2017). The communication style also appears to be closely related to the person’s concept of Self: individuals who perceive themselves as independent from others can communicate in a more assertive way than other individuals who present a concept of Self that is more interdependent (Kirtley and Weaver, 2010). Considering communication style as a specific form of adaptation of the person constitutes a way to adequately integrate the behavioral and personality perspectives (Waldherr and Muck, 2011; Ntoumanis et al., 2017; Tomášková, 2018).

De Vries has progressively perfected his inventory of communication styles through studies in which he tested the convergent validity of the instrument with the measurements of personality (De Vries et al., 2013). His analyses showed strong and medium level associations with the communication styles, thus further validating the integration between the perspectives of traits and communication styles.

The *Communication Styles Inventory* consists of 96 items which refer to the communicative behavior of the person. The items are equally divided among six scales (16 items per scale): *Expressivity*, *Precision*, *Verbal Aggressiveness*, *Critical Spirit*, *Emotionality*, *Impression Manipulation*. Each scale is composed of four sub-scales, each including four items. All the items must receive a reply on the Likert scale with an interval of five points (from 1 = complete disagreement to 5 = total agreement). The Cronbach reliability of the scale oscillates from 0.82 to 0.88 in the study where a non-student sample was used; from 0.83 to 0.87 with a sample composed of students (De Vries et al., 2013).

Considering the fact that the Italian context does not yet have a homologous instrument for measuring communication styles, we have seen fit to develop and present in this paper a preliminary validation study for an Italian version of the *Communication Styles Inventory (CSI-B).*

## Materials and Methods

### Linguistic Procedures

The translation of the CSI followed forward and backward translations of the original scale, following the EORTC translation guidelines (Dewolf et al., 2009). Two Italian translators independently completed the forward translation and negotiated any differences in the two versions. The reconciled Italian version was then given to two English translators, who independently back-translated the measure. Any discrepancies were discussed and resolved, and modifications were made in the CSI to take into account any rewording to improve the conceptual relevance and comprehension of the items. Finally, a small focus group of five university students was convened, the resulting Italian CSI was administered and, based on the discussion of each item, final and minor modifications were made.

### Participants and Administration Procedure

For the purposes of CFA analysis to test the psychometric adequacy of a short form of the original De Vries instrument, the scale was administered to a sample of 1,230 participants (447 males and 783 females with an average age of 31.42 and SD = 10.67. The concurrent validity was tested through a second sample of participants consisting of 518 individuals (228 males and 290 females) with an average age of 26.40 and SD = 9.50. All of them accepted voluntarily to participate in the study after being informed of its objectives and they all supplied an adequate compilation of the instrument. They were also informed of the anonymity of the test and the fact that it was designed for research purposes only. The protocol was approved by Institutional Review Board of the University of Cassino and Southern Lazio. The recruitment phase was carried out between the months of November 2019 and January 2020.

### Measures

*Communication Styles Inventory* (CSI) (De Vries et al., 2013) 96 items articulated in six scales (16 items per scale): *Expressivity*, *Precision*, *Verbal Aggressiveness*, *Critical Spirit*, *Emotionality*, *Impression Manipulation*. Each scale was composed of four sub-scales, each including four items. Likert scale with an interval of five points (from 1 = complete disagreement to 5 = total agreement).

*Multidimensional Personality Profile* (MPP) (Caprara et al., 2006): the test was developed from the two dominant models in the psychology of personality: the social-cognitive theory and the theory of traits. The test measures five fundamental areas of personality: Agentivity, Social-Emotional Intelligence, Self-regulation, Ability to Cope (with critical situations), Innovation. To these is added the evaluation of further personal skills and characteristics, such as Self-esteem, Social Desirability, Cynicism, Impression Management. Each of these areas is divided into sub-dimensions that analyze its content by anchoring it directly to behaviors, subjective states and significant external criteria. The test consists of 152 statements with which it is necessary to express one’s degree of agreement on a five-level scale.

### Statistical Analysis

The sample size was based on the ability to verify an adequate fit of CSI starting with a translation of the full English version that included a six-factor model with 96 manifest variables. Using the root-mean-square error of approximation (RMSEA) as the measure of model fit, a minimum of 960 participants provides a 90% power level to test RMSEA ≤ 0.05 when RMSEA = 0.08, using a 0.05 significance level (MacCallum et al., 1996). The main statistical analyses carried out were the following: verification of the assumptions of univariate and multivariate normality; explorative factorial analysis (EFA) with Parallel Analysis (PA), Maximum Likelihood (ML), and Principal Axis Factoring (PAF) as extraction methods, Promax rotation; Confirmatory Factorial Analysis (CFA); assessment of internal consistency through Cronbach’s alpha coefficient and McDonalds ω; evaluation of significance of correlation coefficients to test concurrent validity of the tool. EFAs were performed according to the methodological recommendations of Goretzko et al. (2019). Statistical analyses were performed using the packages SPSS version 22, JASP 0.12.2, and IBM Amos Graphics 18.

To test the adequacy of the model the following ten indices were considered: (1) chi square; (2) the relationship between the chi-square value and the degrees of freedom (*χ*^2^/d.f., values between 1 and 3 are considered acceptable); (3) GFI (*Goodness of Fit Index*), with values higher than 0.90 indicating an acceptable fit of the model, while a good fit with values higher than 0.95; (4) AGFI (*Adjusted Goodness of Fit Index*), with values higher than 0.90 indicating an acceptable fit of the model, while a good fit with values higher than 0.95; (5) RMSEA (*Root-Mean-Square Error of Approximation*), with values between 0.05 and 0.8 indicating an acceptable fit of the model, while a good fit with values lower than 0.05; (6) *p-value for the test of close fit*, with values between 0.50 and 1 indicating an acceptable fit of the model, while a good fit with values between 0.05 and 0.50; (7) CFI (*Comparative Fit Index*), with values between 0.95 and 0.97 indicating an acceptable fit of the model, while a good fit with values between 0.97 and 1; (8) NFI (*Normed Fit Index*), with values between 0.90 and 0.95 indicating an acceptable fit of the model, while a good fit with values between 0.95 and 1 (Infante and Wigley, 1986; Hu and Bentler, 1999; Byrne, 2001; Schermelleh-Engel et al., 2003; Barbaranelli and Ingoglia, 2013) (9) PNFI (*Parsimony Normed Fit Index*), with values between 0.50 and 0.60 indicating an acceptable fit of the model, while a good fit with values between 0.60 and 1; (10) PCFI (*Parsimony Comparative Fit Index*), with values between 0.50 and 0.60 indicating an acceptable fit of the model, while a good fit with values between 0.60 and 1 (Infante and Rancer, 1982; Mulaik et al., 1989).

Concurrent validity was determined by comparing the correlations between the Communication Styles Inventory factors and the five factors that make up the MPP personality measurement model. To measure concurrent validity, Pearson coefficients were computed.

## Results

The verification of the assumptions of univariate and multivariate normality has been conducted using the procedure for the standardization of the variables, erasing the outlier cases with values greater than 3, then secondly, after calculating the Mahalanobis Distance, eliminating the multivariate outlier cases with D^2^ greater than the critical value, calculated by considering chi-square as the reference distribution (level *p* < 0.001) with p degrees of liberty equal to the number of variables (Cattell and Vogelmann, 1977; Barbaranelli, 2006). The calculation of the Mardia Index (average of the squares of the Mahalanobis Distances) produced a coefficient (9285.81) lower than the limit value (9408). This selection of cases from the original matrix implied the elimination of 186 participants. Therefore, the rest of the validation procedure was carried out with 1,044 cases, 360 of which were males (34.5%) and 684 females (65.5%). The average age was 31.45 with DS = 10.37.

The evaluation of the metric properties of the scale was conducted both through an explorative factor analysis (EFA) and a confirming analysis (CFA) designed to test the goodness of the multidimensional model adopted by De Vries et al. (2013). The averages and standard deviations for the single items and those differentiated by gender are reported in Table 1.

**TABLE 1:** Averages and standard deviations differentiated by gender.

	**Sample (*N* = 1,044)**		**Males (*N* = 360)**		**Females (*N* = 684)**		***t*-test**
**Item**	**Mean**	**SD**	**Mean**	**SD**	**Mean**	**SD**	***t***
Item 1	3.29	0.94	3.28	0.84	3.29	0.98	–0.10
Item 2	3.79	0.70	3.82	0.70	3.78	0.69	0.51
Item 3	3.24	1.13	3.11	1.14	3.31	1.12	–1.59
Item 4	3.16	1.08	3.13	1.12	3.18	1.06	–0.382
Item 5	3.05	1.11	2.70	0.91	3.23	1.16	−4.66***
Item 6	2.09	1.01	2.03	1.04	2.11	1.00	–0.70
Item 7	3.48	0.88	3.58	0.87	3.43	0.89	1.50
Item 8	3.73	0.96	3.89	0.85	3.64	1.00	2.42*
Item 9	3.06	0.94	2.92	0.91	3.14	0.94	−2.12*
Item 10	2.59	0.98	2.53	1.02	2.61	0.96	–0.73
Item 11	3.20	1.07	3.15	1.06	3.22	1.08	–0.57
Item 12	2.45	1.13	2.55	1.12	2.40	1.14	1.18
Item 13	2.92	1.02	3.12	1.01	2.81	1.02	2.67*
Item 14	2.88	0.86	2.98	0.91	2.83	0.82	1.60
Item 15	4.17	0.88	4.12	0.93	4.20	0.86	–0.81
Item 16	3.82	0.79	3.80	0.76	3.83	0.81	–0.37
Item17	3.31	1.10	3.17	1.11	3.39	1.09	–1.77
Item 18	2.92	1.05	3.02	0.96	2.87	1.10	1.21
Item 19	2.26	0.89	2.31	0.90	2.24	0.89	0.70
Item 20	3.49	0.85	3.48	0.84	3.50	0.85	0.30
Item 21	4.13	0.77	4.01	0.80	4.20	0.74	−2.20*
Item 22	3.33	0.89	3.46	0.80	3.26	0.93	2.00*
Item 23	3.16	1.01	2.94	1.06	3.27	0.96	−2.93**
Item 24	2.59	1.00	2.71	0.96	2.53	1.02	1.61
Item 25	3.13	1.11	3.03	1.04	3.18	1.14	–1.17
Item 26	2.76	1.01	2.75	1.06	2.77	0.98	–0.19
Item 27	3.25	1.11	3.43	1.05	3.16	1.13	−−2.22*
Item 28	3.01	0.87	3.02	0.91	3.00	0.85	0.17
Item 29	3.32	0.96	2.98	0.94	3.50	0.93	−4.95***
Item 30	2.22	0.99	2.18	0.93	2.24	1.03	–0.52
Item 31	2.82	0.81	2.77	0.77	2.85	0.83	–0.87
Item 32	3.61	0.90	3.68	0.84	3.57	0.93	1.07
Item 33	2.96	1.05	3.09	0.98	2.89	1.08	1.67
Item 34	3.52	0.94	3.55	0.92	3.50	0.95	0.47
Item 35	3.17	1.02	3.02	1.00	3.25	1.02	−2.04*
Item 36	2.67	1.14	3.07	1.12	2.46	1.10	4.90***
Item 37	2.17	0.80	2.14	0.86	2.18	0.77	–0.42
Item 38	2.88	0.96	2.88	0.98	2.89	0.95	–0.10
Item 39	2.41	1.09	2.75	1.14	2.23	1.03	4.30***
Item 40	3.36	1.02	3.53	1.00	3.27	1.03	2.32*
Item 41	2.94	0.98	2.69	1.02	3.07	0.93	−3.43**
Item 42	2.41	0.96	2.39	0.94	2.43	0.97	–0.31
Item 43	3.30	0.87	3.35	0.78	3.28	0.91	0.75
Item 44	3.39	0.85	3.41	0.92	3.39	0.81	0.23
Item 45	4.09	0.72	4.03	0.68	4.13	0.75	–1.30
Item 46	2.68	1.02	2.79	0.95	2.62	1.04	1.51
Item 47	3.13	1.12	3.31	1.12	3.03	1.11	2.21*
Item 48	2.90	0.98	2.97	1.06	2.86	0.93	0.97
Item 49	2.56	1.05	2.42	1.02	2.64	1.05	–1.90
Item 50	3.53	0.75	3.54	0.84	3.53	0.69	0.18
Item 51	3.32	0.93	3.23	0.90	3.36	0.95	–1.25
Item 52	2.85	0.89	2.93	0.89	2.81	0.88	1.26
Item 53	3.68	0.94	3.35	1.01	3.86	0.86	−4.67***
Item 54	2.51	1.01	2.68	1.03	2.43	0.99	2.28*
Item 55	3.01	0.85	3.12	0.91	2.96	0.82	1.62
Item 56	2.83	0.99	2.84	1.03	2.83	0.98	0.11
Item 57	2.61	1.05	2.72	1.06	2.55	1.04	1.39
Item 58	2.72	1.04	2.62	1.04	2.78	1.03	–1.40
Item 59	3.45	0.94	3.19	0.90	3.58	0.93	−3.74***
Item 60	3.74	1.11	3.44	1.09	3.89	1.09	−3.69***
Item 61	3.20	0.82	3.39	0.77	3.11	0.83	3.14**
Item 62	3.41	0.84	3.45	0.83	3.39	0.84	0.68
Item 63	2.21	1.02	2.36	1.05	2.13	1.00	2.02*
Item 64	3.39	0.94	3.46	0.91	3.36	0.96	0.97
Item 65	3.34	0.98	3.60	0.89	3.21	1.01	3.61***
Item 66	3.07	0.98	3.35	0.98	2.92	0.94	3.97***
Item 67	3.49	0.81	3.66	0.78	3.39	0.81	2.92**
Item 68	2.89	0.90	2.98	0.88	2.83	0.90	1.48
Item 69	4.08	0.71	4.06	0.66	4.10	0.74	–0.47
Item 70	3.43	0.92	3.38	0.94	3.46	0.91	–0.86
Item 71	3.15	1.01	2.88	0.94	3.29	1.02	3.68***
Item 72	3.49	0.94	3.46	0.87	3.51	0.97	–0.48
Item 73	3.28	1.03	3.29	0.92	3.27	1.08	0.21
Item 74	3.57	0.74	3.61	0.73	3.55	0.75	0.72
Item 75	3.19	0.98	3.12	1.02	3.22	0.96	–0.97
Item 76	3.07	0.96	3.17	0.94	3.02	0.97	1.34
Item 77	3.36	0.96	2.99	0.98	3.55	0.89	−5.40***
Item 78	2.69	0.98	2.76	0.95	2.65	0.99	0.95
Item 79	3.11	0.84	3.23	0.87	3.05	0.82	1.92
Item 80	3.52	0.82	3.68	0.76	3.43	0.84	2.62*
Item 81	2.38	0.90	2.54	0.91	2.30	0.89	2.41*
Item 82	3.13	0.92	3.08	0.93	3.16	0.92	–0.80
Item 83	3.29	1.00	3.04	0.97	3.42	0.99	−3.38**
Item 84	2.47	1.07	2.64	1.00	2.38	1.10	2.16*
Item 85	3.49	0.88	3.56	0.85	3.45	0.90	1.12
Item 86	2.98	0.96	3.02	0.95	2.96	0.97	0.56
Item 87	1.85	0.97	2.08	1.02	1.73	0.93	3.25**
Item 88	3.29	0.88	3.32	0.90	3.28	0.87	0.40
Item 89	3.28	1.08	3.04	1.07	3.41	1.07	−3.03**
Item 90	2.99	0.94	2.82	0.93	3.08	0.94	−2.52*
Item 91	2.49	0.88	2.41	0.78	2.53	0.92	1.19
Item 92	3.47	0.77	3.48	0.74	3.46	0.78	0.11
Item 93	4.24	0.72	4.13	0.77	4.30	0.68	−2.05*
Item 94	3.16	0.86	3.05	0.85	3.22	0.86	–1.75
Item 95	2.89	0.96	2.71	0.90	2.99	0.97	−2.65*
Item 96	3.68	0.95	3.48	0.93	3.78	0.95	−2.75*

Item number relates to CSI 2013. SD = Standard Deviation. * *p* < 0.05; ** *p* < 0.005; *** *p* < 0.0005.

An Exploratory Factor Analysis (EFA) with PA and estimation method of ML was conducted on the 96 items of the first version of the questionnaire, producing a Kaiser-Meyer-Olkin (KMO) index score of 0.815, a statistically significant Bartlett’s test (*p* < 0.001) a Chi-squared Test <0.001, RMSEA = 0.040, RMSEA TLI = 0.83. Considering Promax as rotation method and Cattell’s scree test indications (that only four main factors lay above the debris), 86 items resulted in the factor loadings Structure Matrix. For comparison purposes, a further PA with an estimation method of PAF was conducted. Once again, Cattell’s scree test indicated four factors above the debris, and 84 items resulted in the factor loadings Structure Matrix.

From the combination of the two estimation methods, before proceeding with the confirmatory analysis, four factors have therefore been reaffirmed and the 84 items common to both factor loadings SMs have been selected. This selection was subjected to a final EFA with ML and manual input of a maximum of four factors. Factor loadings indicated the elimination of eleven more items, arriving at a final number of 73 items. Kaiser-Meyer-Olkin (KMO) index score was 0.820, Bartlett’s test *p* < 0.001; Chi-squared Test <0.001; RMSEA = 0.055, RMSEA 90% confidence 0.04 - na; TLI = 0.66. Loadings pattern matrix is reported in Table 2.

**TABLE 2:** EFA loadings pattern matrix sorted by dimension.

	**Factor 1**	**Factor 2**	**Factor 3**	**Factor 4**	**Uniqueness**
Item 17		0.485			0.671
Item 18					0.871
Item 19			−0.461		0.801
Item 20				0.342	0.889
Item 21				0.528	0.660
Item 22					0.815
Item 23		0.548			0.690
Item 24	0.588				0.602
Item 25			0.383		0.766
Item 26			−0.419		0.790
Item 27					0.767
Item 28					0.755
Item 29		0.586			0.658
Item 30	0.499				0.649
Item 31			−0.415		0.766
Item 32				0.791	0.467
Item 33	0.475				0.725
Item 34					0.845
Item 35		0.491			0.721
Item 36	0.639				0.604
Item 37			0.454		0.777
Item 41		0.517			0.596
Item 42			−0.533		0.674
Item 43				0.477	0.765
Item 44				0.435	0.791
Item 45				0.337	0.713
Item 46	0.562				0.595
Item 47		0.370			0.765
Item 48	0.508				0.714
Item 49			−0.629		0.620
Item 52	0.333				0.815
Item 53		0.572			0.689
Item 54	0.697				0.540
Item 57	0.491				0.765
Item 58			0.379		0.849
Item 59		0.626			0.627
Item 60	−0.449				0.780
Item 61			0.390		0.635
Item 63	0.364				0.823
Item 64			0.314		0.648
Item 65			0.418		0.625
Item 66		−0.310			0.849
Item 67			0.516		0.567
Item 69				0.390	0.626
Item 71		0.561			0.668
Item 72	−0.428				0.726
Item 73			0.568		0.622
Item 74				0.405	0.783
Item 75		0.308			0.807
Item 76	0.432				0.670
Item 77		0.667			0.564
Item 78	0.566				0.614
Item 79	0.379				0.655
Item 80				0.717	0.544
Item 81	0.498				0.751
Item 82			0.348		0.749
Item 83		0.689			0.556
Item 84	0.686				0.554
Item 87	0.481				0.773
Item 88				0.313	0.724
Item 89		0.602			0.547
Item 90		0.329			0.894
Item 91			−0.448		0.722
Item 92				0.409	0.813
Item 94				0.305	0.719
Item 95			−0.369		0.728
Item 14					0.788
Item 40					0.821
Item 51					0.885
Item 62					0.885
Item 68					0.830
Item 70					0.763

*Extraction Method: Maximum Likelihood. Rotation Method: Promax with Kaiser Normalization. Rotation converged in 11 iterations. Item number relates to CSI 2013.*

Considering the saturations obtained and comparing with the subscales of the CSI items congruent with the content have been gathered on each component and reported in Table 3.

**TABLE 3:** EFA loadings pattern matrix sorted by dimension.

	**Factor 1**	**Factor 2**	**Factor 3**	**Factor 4**
Item 54	**0.697**	−0.054	−0.057	−0.003
Item 84	**0.686**	−0.099	0.012	0.016
Item 12	**0.656**	−0.199	0.072	−0.080
Item 36	**0.639**	−0.096	0.060	−0.003
Item 24	**0.588**	0.044	−0.293	0.003
Item 78	**0.566**	0.110	−0.222	−0.067
Item 46	**0.562**	−0.038	0.237	0.022
Item 6	**0.513**	0.072	−0.268	−0.054
Item 48	**0.508**	−0.055	−0.281	0.129
Item 30	**0.499**	0.081	−0.335	−0.052
Item 81	**0.498**	0.001	−0.032	0.039
Item 57	**0.491**	−0.024	0.022	−0.075
Item 87	**0.481**	−0.034	0.004	−0.123
Item 33	**0.475**	0.046	0.142	−0.021
Item 60	−**0.449**	0.284	0.024	0.132
Item 76	**0.432**	0.097	0.260	0.028
Item 72	−**0.428**	0.256	0.254	0.159
Item 79	**0.379**	−0.008	0.364	0.071
Item 63	**0.364**	0.007	0.183	−0.054
Item52	**0.333**	0.095	0.124	0.073
Item 4	**0.322**	0.164	0.211	−0.101
Item 83	−0.116	**0.689**	0.070	−0.051
Item 77	−0.061	**0.667**	0.099	−0.022
Item 59	−0.077	**0.626**	0.009	−0.109
Item 89	0.006	**0.602**	−0.326	0.032
Item 29	−0.060	**0.586**	0.126	−0.026
Item 53	−0.077	**0.572**	0.042	0.004
Item 71	−0.071	**0.561**	−0.133	−0.123
Item 23	0.023	**0.548**	−0.068	−0.017
Item 41	0.037	**0.517**	−0.381	0.016
Item 5	−0.159	**0.505**	−0.023	0.015
Item 35	0.087	**0.491**	0.071	−0.030
Item 17	0.097	**0.485**	−0.278	0.017
Item 3	0.121	**0.386**	0.059	−0.066
Item 11	0.130	**0.372**	−0.146	0.045
Item 47	0.040	**0.370**	−0.103	−0.278
Item 90	−0.059	**0.329**	0.075	−0.009
Item 66	0.221	−**0.310**	−0.055	0.234
Item 75	0.229	**0.308**	−0.018	−0.132
Item 49	−0.078	0.156	−**0.629**	0.127
Item 73	0.172	0.178	**0.568**	−0.170
Item 7	0.293	−0.025	**0.545**	0.064
Item 42	0.209	0.161	−**0.533**	0.031
Item 67	0.151	−0.201	**0.516**	0.181
Item 13	0.459	−0.046	**0.463**	−0.085
Item 19	0.182	−0.121	−**0.461**	0.161
Item 37	0.057	−0.117	**0.454**	0.001
Item 91	0.157	0.274	−**0.448**	0.104
Item 26	0.125	0.198	−**0.419**	0.069
Item 65	0.090	−0.332	**0.418**	0.198
Item 31	−0.023	0.300	−**0.415**	0.109
Item 1	0.085	0.202	**0.409**	0.000
Item 61	0.332	0.100	**0.390**	0.074
Item 25	0.176	0.207	**0.383**	−0.139
Item 58	0.010	−0.070	**0.379**	0.012
Item 95	0.077	0.365	−**0.369**	0.010
Item 10	−0.056	0.099	**0.352**	−0.050
Item 82	−0.049	0.308	**0.348**	0.096
Item 64	0.169	0.186	**0.314**	0.266
Item 32	0.021	−0.112	−0.263	**0.791**
Item 80	0.007	−0.115	−0.145	**0.717**
Item 8	−0.096	−0.126	−0.241	**0.680**
Item 21	−0.128	−0.046	0.117	**0.528**
Item 43	0.108	0.018	−0.357	**0.477**
Item 44	−0.011	−0.231	−0.071	**0.435**
Item 16	−0.037	0.211	0.111	**0.424**
Item 92	−0.034	−0.111	0.041	**0.409**
Item 74	−0.078	−0.050	0.125	**0.405**
Item 69	−0.195	0.252	0.275	**0.390**
Item 20	−0.050	−0.091	−0.058	**0.342**
Item 45	−0.158	0.313	0.176	**0.337**
Item 88	0.059	0.121	0.270	**0.313**
Item 94	0.253	0.212	0.057	**0.305**

*Extraction Method: Maximum Likelihood. Rotation Method: Promax with Kaiser Normalization. Rotation converged in 11 iterations. Item number relates to CSI 2013. Bold indicates the groupings of factors ordered by size.*

As can be observed in Table 3, the first factor comprised the subscales that De Vries indicated as *Ingratiation*, *Charm* and *Authoritarianism*. We referred to the underlying main factor that brought them together as ***Impression Manipulativeness***. The second factor included the subscales of *Worrisomeness*, *Sentimentality*, *Tension* and *Defensiveness*. We indicated this underlying factor as ***Emotionality***. The third factor presented the aggregation of *Talkativeness*, *Conversational Dominance, Humor* and *Philosophicalness*. The underlying factor that brought them together has been indicated as ***Expressiveness***. The fourth factor presented the aggregation of *Inquisitiveness, Thoughtfulness, Non-supportiveness, and Conciseness* with an underlying factor which was called ***Preciseness.***

In order to examine the validity of a 73-item CSI construct, a CFA was performed, using the Amos Graphics 18 software. The results obtained considering four factors did not show a good adaptation to the data. Therefore, the existence of a lower number of factors was verified. A further EFA with ML and manual input of maximum three factors was performed. Factor loadings indicated the elimination of thirty more items, arriving at a final number of 48 items. Kaiser-Meyer-Olkin (KMO) index score was 0.820, Bartlett’s test *p* < 0.001; Chi-squared Test < 0.001; RMSEA = 0.06; TLI = 0.58. Pattern matrix is reported in Table 4.

**TABLE 4:** EFA loadings pattern matrix sorted by dimension (three factors).

	**Factor 1**	**Factor 2**	**Factor 3**
Item 69	**0.574**	−0.218	0.194
Item 67	**0.561**	0.140	−0.275
Item 7	**0.548**	0.290	−0.101
Item 64	**0.527**	0.144	0.139
Item 88	**0.504**	0.031	0.077
Item 82	**0.466**	−0.055	0.247
Item 45	**0.458**	−0.178	0.271
Item 21	**0.451**	−0.165	−0.070
Item 49	−**0.449**	−0.098	0.246
Item 65	**0.448**	0.078	−0.390
Item 61	**0.442**	0.326	0.047
Item 73	**0.441**	0.192	0.090
Item 70	**0.437**	0.120	0.107
Item 16	**0.437**	−0.069	0.185
Item 42	−**0.424**	0.194	0.247
Item 1	**0.422**	0.090	0.137
Item 79	**0.403**	0.370	−0.052
Item 54	−0.055	**0.690**	−0.021
Item 84	0.008	**0.679**	−0.078
Item 12	−0.026	**0.659**	−0.185
Item 36	0.041	**0.635**	−0.084
Item 24	−0.246	**0.575**	0.109
Item 78	−0.216	**0.562**	0.161
Item 46	0.243	**0.553**	−0.056
Item 6	−0.260	**0.508**	0.131
Item 57	−0.025	**0.491**	−0.011
Item 30	−0.316	**0.490**	0.149
Item 81	0.013	**0.485**	0.023
Item 87	−0.071	**0.482**	−0.018
Item 48	−0.167	**0.480**	0.007
Item 33	0.139	**0.471**	0.039
Item 13	0.365	**0.465**	−0.102
Item 60	0.174	−**0.457**	0.260
Item 72	0.393	−**0.431**	0.197
Item 76	0.298	**0.424**	0.068
Item 83	0.177	−0.104	**0.662**
Item 89	−0.141	−0.001	**0.641**
Item 77	0.225	−0.054	**0.639**
Item 59	0.066	−0.061	**0.610**
Item 71	−0.090	−0.055	**0.571**
Item 41	−0.218	0.030	**0.567**
Item 29	0.227	−0.051	**0.554**
Item 53	0.163	−0.071	**0.552**
Item 23	0.044	0.028	**0.551**
Item 17	−0.127	0.090	**0.522**
Item 5	0.097	−0.155	**0.494**
Item 35	0.158	0.089	**0.474**
Item 95	−0.251	0.072	**0.419**

*Extraction Method: Maximum Likelihood. Rotation Method: Promax with Kaiser Normalization. Rotation converged in 7 iterations. Item number relates to CSI 2013. Bold indicates the groupings of factors ordered by size.*

**FIGURE 1:**
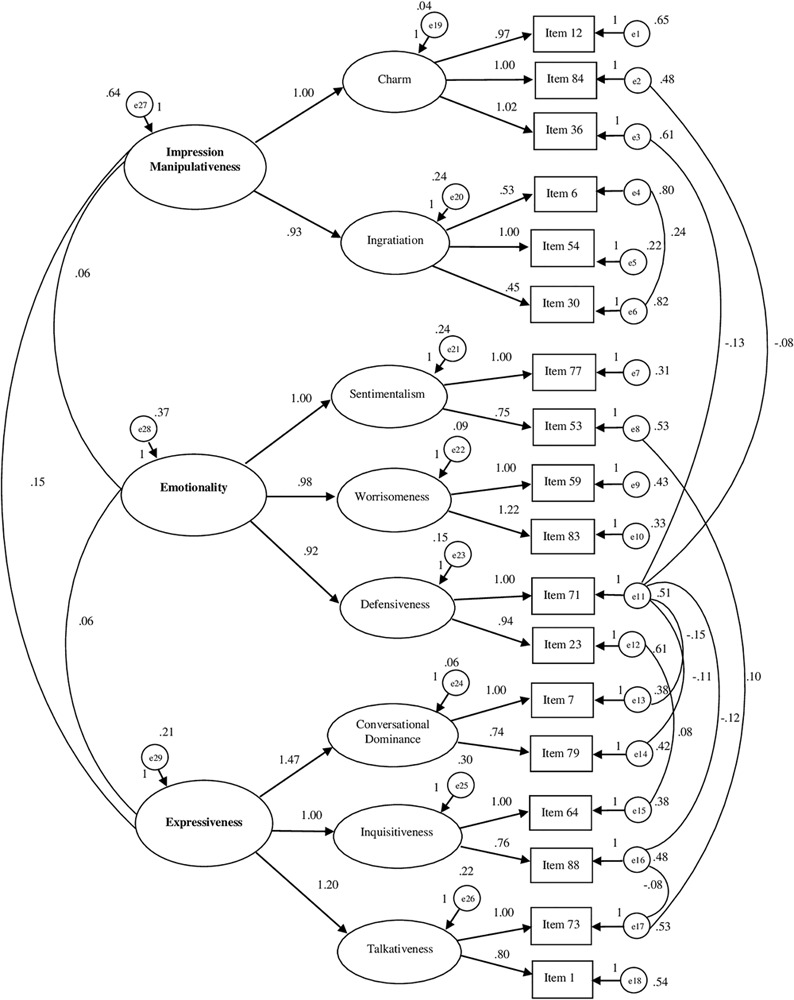
Path diagram of the confirmatory analysis concerning CSI-B/I (18 items). χ2/df = 1.251; RMSEA = 0.027; RMSEA 90% CI = 0.008–0.040; GFI = 0.958; AGFI = 0.937; CFI = 0.983; NFI = 0.922.

The CFA bore out that the model with three related factors presented overall good indices of adaptation to data (see Table 5 and Figure 1). Overall, the model included 18 items. The first factor measures *Impression Manipulativeness* (6 items) and includes ingratiation and charm. The second factor measures the *Emotionality* (6 items) and includes sentimentality, worrisomeness and defensiveness. The third factor measures the *Expressiveness* (6 items) and includes talkativeness, conversational dominance and inquisitiveness.

**TABLE 5:** Fit indices of confirmatory factor analysis (three-factor Model).

	***X*^2^**	***p***	**df**	***X*^2^/df**	**GFI**	**AGFI**	**RMSEA**	**RMSEA 90% CI**	***p*-close**	**CFI**	**NFI**	**PNFI**	**PCFI**
*N* = 1044	143.90	0.035	115	1.251	0.958	0.937	0.027	0.008–0.040	0.999	0.983	0.922	0.693	0.739
Fit measure				good	good	acceptable	good	good	good	good	acceptable	good	good

Finally, a further EFA with ML on the list of the 18 items. Table 6 shows the model matrix with saturations on the three identified factors, McDonald’s ω and Crombach’s Alpha values, Guttman Split-Half Coefficients, Corrected item/total correlations.

**TABLE 6:** Pattern matrix EFA (18 items).

	**Impression Manipulativeness**	**Emotionality**	**Expressiveness**
Item 54	**0.745**	0.050	0.026
Item 84	**0.716**	−0.034	0.086
Item 12	**0.691**	−0.101	0.042
Item 36	**0.648**	−0.078	0.111
Item 6	**0.516**	0.132	−0.138
Item 30	**0.497**	0.160	−0.216
Item 83	−0.033	**0.730**	0.058
Item 59	−0.026	**0.662**	0.060
Item 71	0.082	**0.642**	−0.195
Item 77	−0.061	**0.629**	0.175
Item 23	0.109	**0.544**	−0.023
Item 53	0.018	**0.534**	0.083
Item 7	0.043	−0.084	**0.749**
Item 79	0.109	−0.069	**0.585**
Item 64	−0.032	0.077	**0.533**
Item 1	−0.078	0.125	**0.520**
Item 88	−0.096	−0.013	**0.501**
Item 73	0.029	0.163	**0.498**
*α*	0.80	0.79	0.74
ω	0.80	0.80	0.74
*λ6*	0.79	0.78	0.73
r*	0.39	0.39	0.32

Extraction Method: Maximum Likelihood. Rotation Method: Promax with Kaiser Normalization. Rotation converged in 5 iterations. Item number relates to CSI 2013. *α*, Crombach’s alpha; ω, McDonald’s omega*; λ6*, Gutmann’s lambda; r*, average inter-item correlation. Bold indicates the groupings of factors ordered by size.

Concurrent validity was tested by examining the significance of correlation coefficients between Communication Styles Inventory factors and the five factors that make up the MPP personality measurement model, relating to a second administration carried out on a sample of 518 individuals (228 males and 290 females) with an average age of 26.40 and SD = 9.50. In relation to the results of this association, three hypotheses have been formulated: (1) the higher the *Impression Manipulativeness*, the higher the *Cynicism and Impression Management* would have been; (2) the higher the *Emotionality in Conversation*, the higher the *Social-Emotional Intelligence* would have been and the lower the *Ability to Cope*; (3) the higher the *Expressiveness*, the higher the *Agentivity* and the *Self-Regulation* would have been. As shown in Table 7, the results have confirmed the assumed directions of correlation; therefore, the measure proved good convergent validity with *Multidimensional Personality Profile* (MPP) by Caprara et al. (2006) and consequently its usefulness in describing communication styles in relation to personality traits. McDonald’s ω and Alpha coefficients for this second administration were 0.80 and 0.80 (*Impression Manipulativeness*), 0.80 and 0.79 (*Emotionality*), 0.74 and 0.74 (*Expressiveness*), respectively.

**TABLE 7:** Correlations of the Italian version of the Communication Styles Inventory-Brief (CBI-B/I) with the Multidimensional Personality Profile (MPP).

		**IMP**	**EM**	**EX**	**AG**	**SEI**	**SR**	**AC**	**IN**	**CY**	**IMG**
CSI	IMP	1									
	EM	0.053	1								
	EX	0.199*	0.147**	1							
MPP	AG	0.146	−0.122**	0.498**	1						
	SEI	–0.096	0.271**	0.335**	0.330**	1					
	SR	–0.085	0.026	0.313**	0.663**	0.292**	1				
	AC	0.022	−0.405**	0.111	0.550**	0.433**	0.440**	1			
	IN	−0.185*	0.202**	0.221**	0.430**	0.310**	0.418**	0.257**	1		
	CY	0.416**	–0.118	0.114	0.191*	−0.285**	–0.064	0.005	–0.144	1	
	IMG	0.283**	0.037	0.498**	0.497**	0.395**	0.367**	0.212*	0.118	0.049	1

IMP, Impression Manipulation; EM, Emotionality; EX, Expressiveness; AG, Agentivity; SEI, Socio-Emotional Intelligence; SR, Self-Regulation; AC, Ability to Cope; IN, Innovation; CY, Cynicism; IMG, Impression Management. ** Correlation is significant at the 0.01 level (2-tailed). * Correlation is significant at the 0.05 level (2-tailed).

The following Table 8 reports English and Italian versions of the CSI-B, and the grouping of the items on respective factors.

**TABLE 8:** Communication Styles Inventory – Brief (CSI-B).

**English version**	**Italian version**
1. I sometimes use my charm to get something done (IM-Charm).	1. Qualche volta uso il mio fascino per ottenere qualcosa.
2. People can tell that I am emotionally touched by some topics of conversation (EM – Sentimentality).	2. Le persone potrebbero dire di me che vengo emotivamente toccato da alcuni argomenti di conversazione.
3. I often take the lead in a conversation (EX – Conversational Dominance).	3. Mi capita spesso di prendere l′iniziativa e condurre il discorso in una conversazione.
4. I sometimes put on a very seductive voice when I want something (IM – Charm).	4. Qualche volta tiro fuori una voce molto seduttiva quando voglio qualcosa.
5. When describing my memories, I sometimes get visibly emotional (EM – Sentimentality).	5. Quando racconto del mio passato qualche volta posso apparire visibilmente emozionato.
6. I often determine the direction of a conversation (EX – Conversational Dominance).	6. Spesso decido io la direzione di una conversazione.
7. I sometimes flirt a little bit to win somebody over (IM –Charm).	7. A volte flirto un po’ per conquistare qualcuno.
8. People can tell when I feel anxious (EM – Worrisomeness).	8. Gli altri possono riconoscere quando io mi sento in ansia.
9. I ask a lot of questions to uncover someone’s motives (EX – Inquisitiveness)	9. Pongo molte domande per scoprire le motivazioni degli altri.
10. I sometimes praise somebody at great length, without being really genuine, in order to make them like me (IM – Ingratiation).	10. Qualche volta esprimo ampie lodi per qualcuno, senza essere realmente sincero, al fine di piacergli.
11. When I worry, everybody notices (EM – Worrisomeness).	11. Quando sono preoccupato tutti se ne accorgono.
12. I always ask how people arrive at their conclusions (EX – Inquisitiveness).	12. Chiedo sempre alle persone come arrivano alle loro conclusioni.
13. Sometimes I use flattery to get someone in a favorable mood (IM – Ingratiation).	13. Qualche volta faccio uso dell’adulazione per provocare in qualcuno un atteggiamento favorevole.
14. When people criticize me, I am visibly hurt (EM – Defensiveness).	14. Quando le persone mi criticano sono visibilmente ferito.
15. I like to talk a lot (EX – Talkativeness)	15. Mi piace parlare molto.
16. In discussions I sometimes express an opinion I do not support in order to make a good impression (IM –Ingratiation).	16. Al fine di fare una buona impressione, nelle discussioni qualche volta esprimo un’opinione alla quale non credo veramente.
17. The comments of others have a noticeable effect on me (EM – Defensiveness).	17. I commenti degli altri hanno un effetto evidente su di me.
18. I always have a lot to say (EX – Talkativeness).	18. Ho sempre molto da dire.

*IM, Impression Manipulativeness; EM, Emotionality; EX, Expressiveness.*

As far as the scoring of the instrument is concerned, the 18 items in total are distributed over three factors, each comprising six items. Every item has a scoring range from 1 (completely disagree) to 5 (completely agree). The scoring calculation will produce, through a summation of the scores of the component items, separate measurements for each factor. Therefore, *Impression Manipulativeness*: 1 + 4 + 7 + 10 + 13 + 16; *Emotionality*: 2 + 5 + 8 + 11 + 14 + 17; *Expressiveness*: 3 + 6 + 9 + 12 + 15 + 18. Each factor can have a total score range from 6 to 30. Based on the distribution of the scores obtained from the normative sample, the cut-off criteria, differentiated by gender, have been identified and reported in the following Table 9.

**TABLE 9:** Scoring directions of CSI-B/I.

**Factor**	**low**	**medium**	**high**	***M***	**DS**	**SE**	**SK**	**SE**	**KU**	**SE**
**Total sample (*N* = 1,044)**										
IM	6–12	13–16	17–30	14.41	4.49	0.24	0.46	0.13	0.17	0.26
EM	6–19	20–22	23–30	20.08	4.11	0.22	−0.27	0.13	0.31	0.26
EX	6–19	20–21	22–30	19.84	3.63	0.19	−0.22	0.13	0.08	0.26
**Males (*N* = 447)**										
IM	6–13	14–17	18–30	15.16	4.39	0.40	0.32	0.22	−0.45	0.44
EM	6–16	17–20	21–30	18.40	4.19	0.38	0.07	0.22	−0.18	0.44
EX	6–19	20–22	23–30	20.16	3.77	0.34	−0.13	0.22	0.003	0.44
**Females (*N* = 783)**										
IM	6–12	13–15	16–30	14.02	4.50	0.30	0.55	0.16	0.58	0.32
EM	6–20	21–22	23–30	20.96	3.79	0.25	−0.39	0.16	0.98	0.32
EX	6–19	20–21	22–30	19.67	3.60	0.24	−0.29	0.16	0.13	0.32

*IM, Impression Manipulation; EM, Emotionality; EX, Expressiveness; M, Mean; SD, Standard Deviation; SE, Standard Error; SK, Skewness; KU, Kurtosis.*

## Discussion

The analyses carried out led to the definition of a scale composed of a total of 18 items, aggregated into eight subscales, which in turn converge separately on three factors. The first factor measures the ability of the person to exercise an effective *Impression Manipulativeness* during the conversation. Two components were also identified in this case: Charm and Ingratiation. Charm is a mixture of attractive appearance, sensuality and inner security. When in a communication one of the interlocutors appeals to one’s charisma or aesthetic attractiveness, one speaks of arguing through one’s personal charm. We usually measure the success of a conversation by how fascinated we are by what the other person has told us. The person who has charm attracts attention and involves people, despite their own will. In all of this, the voice is an important vehicle of suggestion that can positively influence the interlocutor and deepen the relationship, creating an empathic atmosphere, fundamental for the successful outcome of the conversation. The expert speaker succeeds in exerting a sometimes hypnotic charm on his listeners, to the extent that they hang on to his voice and his words. The ingratiation in the conversation refers to the idea of a conscious expressive choice, of a rational strategy aimed at achieving goals, and which consists in captivating the interlocutor, almost appeasing or seducing him. This mode of communication is based on the pleasure of others, so that they are well prepared to accept the requests that are addressed to them. Individuals need to keep other people’s impressions in line with the perceptions they wish to convey, so as to determine important consequences on the perception and evaluation of others and influence interpersonal dynamics.

The second factor, indicated as *Emotionality*, refers to the emotional activation produced in the individual as a result of verbal interaction. Three distinct components were identified. The first (*Sentimentality*) indicates the emotional transport that tends to accompany the person’s conversation, which with difficulty contains their emotions, both when the subject is current and when it relates to stories that refer to the person’s past. The individual has a pronounced empathic capacity, so that the intense emotional states of others do not leave him/her indifferent, rather he/she tends to try to identify with the emotions of others. The individual is aware of being particularly “sensitive” toward certain topics in particular which emotionally touch him/her more closely. The second component, called the *Defensiveness*, indicates the level of vulnerability perceived by the person following criticisms or verbal attacks received from the interlocutors. Negative comments can cause concern and an immediate closing reaction. The person feels hurt and is aware that he/she will need time to overcome the attacks and criticisms received. The most sensitive individuals tend to assume an acquiescent role in the conversation, in order to avoid situations of blatant opposition, or they prefer to listen in silence, minimizing participation in the speech. The third component was called *Worrisomeness* and refers specifically to the incapacity of the person to mask his/her emotional state in verbal interaction; therefore, the individual fears that every negative emotional state will be immediately grasped by the interlocutor.

The third factor, referred to as *Expressiveness*, refers to the individual’s ability to be effective in conversation, by monitoring and balancing the elements of communication, such as the quality of the topic illustrated, supported with an adequate amount and variety of data and sources; the clear and consistent presentation of the topic to keep interest and attention alive; the enhancement of non-verbal resources, such as posture, gestures, eye contact, pauses and silences; the ability to reposition the speech if it deviates and not least a shrewd management of the time available. Three specific characteristics that define this ability were identified: the disposition or tendency to talk a lot and willingly; choosing and addressing the topics of the conversation; involving the interlocutor with many questions aimed at testing the validity of his opinions.

The positive loquacity of an expressive style serves to better explain concepts and ideas, and is typical of the eloquent person, that is, able to express himself with verbal richness and linguistic knowledge and illustrate even complex concepts with his own words. The above mentioned loquacity can be a “fixed” characteristic of a person, something that is fixed in character, or an occasional behavior: for example, some who becomes loquacious suddenly, because he feels uncomfortable, or instrumentally, because he thinks that in a certain context such behavior is beneficial to him. Those who show a low level of loquacity, on the other hand, might show good listening skills, but also have difficulty expressing their ideas, expressing their desires and intervening in discussions.

Expressive assertiveness is also manifested as conversational dominance, exercising control over the arguments brought into discussion and with the ability to impose one’s own point of view. In this case, the person activates moves that initiate the sequence, i.e., questions, statements through which the subsequent development of the interaction is determined. He therefore exercises control over the topics under discussion, their articulation into sub-themes, their development and their conclusion. In cases where the participants’ agreement on the definition of the current situation seems to be lacking, he has the power to re-establish the interactional order through meta-communicative comments that redefine the contextual framework and the type of activity in which one is involved. Those who instead have low levels of dominance in the conversation tend to play a passive role and adapt to the arguments proposed by others.

The active and reflective attitude of the person during the conversation is also manifested through the tendency to ask the interlocutor precise questions about the content of the conversation, even if they are strict about the truthfulness and plausibility of the conclusions presented. The attention required of the interlocutor to the thematic focus is always high. This may reveal a tendency to not get lost in generic, superficial issues, not considered functional to the objectives of the conversation. A low level of inquisitiveness, on the other hand, may indicate a limit in the critical and reflective capacity of the person, who is substantially disengaged and little involved in the topic of discussion.

The tool altogether allows to evaluate three crucial dimensions that concur jointly with an adaptive exercise of the communicative interaction: the control of the Other (measure of impression manipulativeness); control of oneself (measure of emotionality); control of the expressive means (measure of expressiveness). Concurrent validity testing showed associations worthy of further investigation: first of all, the *Impression Manipulation* factor has shown a significant correlation with the MPP sub-scale of cynicism, which highlights the instrumental aspect of manipulation, the tendency to put one’s own interests before those of others, and to bend others and circumstances to one’s own ends and objectives. The further negative correlation with Innovation is consistent with a profile that is not very open to values, principles and behavioral habits that are different from one’s own, and which is above all concerned with concreteness in situations. Finally, the positive correlation with the MPP subscale of impression management confirms the tendency to use communication tools, using charm and ingratiating to obtain appreciation, esteem and trust.

The predictive measure of *Emotionality* could provide in the first place useful inferences on the dimension of Emotional Intelligence of the person, by virtue of the positive association found, i.e., on the individual abilities that manifest themselves in knowing how to put themselves in other people’s shoes and in knowing how to recognize and express emotions, in knowing how to take into account the needs and requirements of others, in knowing how to put others at ease. At the same time, the inverse relationship with Ability to Cope would allow to infer the measure of the vulnerability of the person in maintaining calm and tranquility in the presence of difficulties and stressful situations, in experiencing states of discomfort and general mood swings, in showing difficulties in recovering after failures, tending to brood over what happened.

The *Expressiveness* factor showed strong positive correlations with the dimensions of Agentivity, Self-regulation and Emotional Intelligence. It would be useful to firstly note the predictive value of the measure of this style of communication on those of Agentivity and Self-Regulation. In the first case, it would mean obtaining useful inferences on the person’s ability to assert his or her own opinions, to set ambitious goals, to know how to guide and motivate others and to conduct one’s activities with vigor and promptness. In the second case, the inferences would concern the individual’s abilities with regard to planning and persistence in achieving a goal, in self-discipline understood as a self-reflexive ability to organize, as well as tenacity in success, love of order and accuracy, which transpires in the desire to do things well. A further positive correlation with emotional intelligence indicates the predictive value of the measure of expressiveness also with respect to the person’s ability to actively listen, to feel at ease with others, and to put others at ease through one’s own behavior. The expressiveness factor therefore demonstrates an important predictive relationship with the assertive capacity and effectiveness in communication.

The tool appears agile and versatile, prefiguring an application during the assessment in multiple contexts where it could be important to obtain information on the communicative attitudes of the person: companies, institutions, staff selection, individual, and group profile analysis, coaching, psychotherapy, counseling, career guidance. Several recent contributions have emphasized the importance of taking into account communication styles in education, health and in the dissemination of news by the media (Ashton et al., 2004; Minnema, 2014; Fourie, 2018; Chlopicki and Laineste, 2019; Labreche et al., 2019; Lõrincz, 2019; Ramachandiran and Mahmud, 2019; Trant et al., 2019; Iskandarova and Griffin, 2020).

## Conclusion

This first Italian validation study of the Communication Styles Inventory turned out to be a significantly reduced version compared to the original instrument of De Vries et al. (2013) which presented 16 scales and six main domains (expressiveness, precision, verbal aggression, critical spirit, emotionality, manipulation of the impression). De Vries’ main objective was to obtain a tool capable of understanding most of the main lexical dimensions together with the behavioral components of the communicative style. The result was a particularly large and composite instrument (96 items). Instead, in our study, starting from the original instrument and through a subsequent analysis of factorial refinement and confirmatory verification, we have achieved a brief tool that displays good fit indices which are suitable for Italians. Future studies could test the adequacy of the model on specific samples of the population and explore the possibility of recovering the dimensions previously excluded in this first work.

## Data Availability Statement

The datasets generated for this study are available on request to the corresponding author.

## Ethics Statement

The studies involving human participants were reviewed and approved by the Institutional Review Board (IRB_SUSS) of the University of Cassino and Southern Lazio. The patients/participants provided their written informed consent to participate in this study.

## Author Contributions

PD, GV, and SM designed the study, analyzed the data, and discussed the results. PD, GV, and AG drafted the manuscript. SM and AG revised the manuscript. All authors approved the final manuscript. Finally, the authors have agreed to be accountable for all aspects of the manuscript in ensuring that questions related to the accuracy or integrity of any part of it are appropriately investigated and resolved.

## Conflict of Interest

The authors declare that the research was conducted in the absence of any commercial or financial relationships that could be construed as a potential conflict of interest.

## Footnotes

1 Dominant, Dramatic, Contentious, Animated, Impression-leaving, Relaxed, Attentive, Open, Friendly.

## References

[B1] Asendorpf J. B. (2004). *Psychologie der Persönlichkeit.* Berlin: Springer-Verlag.

[B2] Ashton M. C. Lee K. Perugini M. Szarota P. de Vries R. Di Blas L. (2004). A six-factor structure of personality-descriptive adjectives: solutions from psycholexical studies in seven languages. *J. Pers. Soc. Psychol.* 86 356–366. 14769090 10.1037/0022-3514.86.2.356

[B3] Bakker-Pieper A. De Vries R. E. (2013). The incremental validity of communication styles over personality traits for leader outcomes. *Hum. Perform.* 26 1–19.

[B4] Barbaranelli C. (2006). *Analisi dei Dati Con SPSS II. Le analisi Multivariate.* Milano MI: LED Edizioni Universitarie.

[B5] Barbaranelli C. Ingoglia S. (2013). *I Modelli di Equazioni Strutturali: Temi e Prospettive.* Milano, MI: LED.

[B6] Beatty M. J. McCroskey J. C. Heisel A. D. (1998). Communication apprehension as temperamental expression: a communibiological paradigm. *Commun. Monogr.* 65 197–219. 10.1080/03637759809376448

[B7] Bechler C. Johnson S. D. (1995). Leadership and listening: a study of member perceptions. *Small Group Res.* 26 77–85.

[B8] Bolton R. Bolton D. G. (1984). *Social Style/Management Style: Developing Productive Work Relationships.* New York, NY: American Management Associations.

[B9] Byrne B. M. (2001). *Structural Equation Modeling with AMOS: Basic Concepts, Applications, and Programming.* Mahwah, NJ: Lawrence Erlbaum Associates.

[B10] Caprara G. V. Barbaranelli C. De Carlo N. A. Robusto E. (2006). *Il Multidimensional Personality Profile (MPP). Un Questionario Di Nuova Generazione Per La Misura Della Personalità.* Milano: Franco Angeli.

[B11] Cattell B. R. Vogelmann S. (1977). A comprehensive trial of the scree and Kg criteria for determining the number of factors. *Multiv. Behav. Res.* 12 289–325. 10.1207/s15327906mbr1203_2 26804294

[B12] Chlopicki W. Laineste L. (2019). Communication styles: between deliberate strategy and ambivalence. *J. Pragmat.* 153 15–19. 10.1016/j.pragma.2019.08.001

[B13] Cole J. G. McCroskey J. C. (2000). Temperament and socio-communicative orientation. *Commun. Res. Rep.* 17 105–114.

[B14] Crews E. R. Brouwers M. Visagie J. (2019). Transformational and transactional leadership effects on communication styles. *J. Psychol. Africa* 29 421–428.

[B15] Croucher S. M. Bruno A. McGrath P. Adams C. McGahan C. Suits A. (2012). Conflict styles and high-low context cultures: a cross-cultural extension. *Commun. Res. Rep.* 29 64–73.

[B16] De Vries R. Bakker-Pieper A. Konings E. F. Schouten B. (2013). The communication styles inventory (CSI): a six-dimensional behavioral model of communication styles and its relation with personality. *Commun. Res.* 40 506–532. 10.1177/0093650211413571

[B17] De Vries R. E. Bakker-Pieper A. Alting Siberg R. Van Gameren K. Vlug M. (2009). The content and dimensionality of communication styles. *Commun. Res.* 36 178–206.

[B18] Dewolf L. Koller M. Velikova G. Johnson C. Scott N. Bottomley A. (2009). *EORTC. Quality of Life Group Translation Procedure*, 3rd Edn, Cambridge, MA: Academic Press.

[B19] Dion P. A. Notarantonio E. M. (1992). Salesperson communication style: the neglected dimension in sales performance. *J. Bus. Commun.* 29 63–77. 10.1177/002194369202900104

[B20] Fourie P. (2018). The communication style of social media communication. *Intern. J. Interdiscipl. Soc. Commun. Stud.* 13 1–10.

[B21] Goretzko D. Pham T. T. H. Bühner M. (2019). Exploratory factor analysis: current use, methodological developments and recommendations for good practice. *Curr. Psychol.* 10.1007/s12144-019-00300-2

[B22] Gudykunst W. B. Matsumoto Y. Ting-Toomey S. Nishida T. Kim K. S. Heyman S. (1996). The influence of cultural individualism-collectivism, self construals, and individual values on communication styles across cultures. *Hum. Commun. Res.* 22 510–543.

[B23] Hansford B. C. Hattie J. (1987). Perceptions of communicator style and self-concept. *Commun. Res.* 14 189–203. 10.1177/009365087014002003

[B24] Harkin J. Davis P. Turner G. (1999). The development of a communication styles questionnaire for use in english 16–19 education. *Westminster Stud. Educ.* 22 31–47.

[B25] Horvath C. W. (1998). “Biological origins of communicator style,” in *Communication Personality: Trait Perspectives*, eds McCroskey J. C. Daly J. A. Martin M. M. (Cresskill, NJ: Hampton Press), 69–94.

[B26] Hu L. T. Bentler P. M. (1999). Cut-off criteria for fit indexes in covariance structure analysis: conventional criteria versus new alternatives. *Struct. Equat. Model. Multidiscipl. J.* 6 1–55. 10.1080/10705519909540118

[B27] Infante D. A. Rancer A. S. (1982). A conceptualization and measure of argumentativeness. *J. Pers. Assess.* 46 72–80. 16370641 10.1207/s15327752jpa4601_13

[B28] Infante D. A. Wigley C. W. (1986). Verbal aggressiveness: an interpersonal model and measure. *Commun. Monogr.* 53 61–69.

[B29] Iskandarova S. Griffin O. (2020). “Assessing multilingual multicultural teachers’ communication styles,” in *Multicultural Instructional Design*, (Berlin: Springer), 1284–1296. 10.4018/978-1-5225-9279-2.ch061

[B30] Kapoor S. Hughes P. C. Baldwin J. R. Blue J. (2003). The relationship of individualism-collectivism and self-construals to communication styles in India and the United States. *Intern. J. Intercul. Relat.* 27 683–700.

[B31] Kirtley M. D. Weaver B. (2010). Exploring the impact of gender role self-perception on communication style. *Women Stud. Commun.* 2 190–209.

[B32] Labreche T. Szilva M. Khan S. (2019). The value of adapting styles of communication in persons with dementia and aphasia seeking ophthalmic care: a case series. *Insight J. Am. Soc. Ophthal. Reg. Nurs.* 44 21–24.

[B33] Leary T. (1957). *Interpersonal Diagnosis Of Personality.* New York, NY: Ronald.

[B34] Leung S. K. Bond M. H. (2001). Interpersonal communication and personality: self and other perspectives. *Asian J. Soc. Psychol.* 4 69–86.

[B35] Liliweri A. (2017). An analysis on the relationship of thinking and learning styles with communication style. *Intern. J. Sch. Cogn. Psychol.* 4:192 10.4172/2469-9837.1000192

[B36] Lõrincz M. (2019). Relationship between communication style and effective teaching. *VYPUSK* 2 147–151. 10.24144/2524-0609.2018.43.147-151

[B37] MacCallum R. Browne M. Sugawara H. (1996). Power analysis and determination of sample size for covariance structure modeling. *Psychol. Methods* 1 130–149.

[B38] McCrae R. R. Costa P. T. Ostendorf F. Angleitner A. Hrebickova M. Avia M. D. (2000). Nature over nurture: temperament, personality, and life span development. *J. Pers. Soc. Psychol.* 78 173–186. 10653513 10.1037//0022-3514.78.1.173

[B39] McCrae R. R. John O. P. (1992). An introduction to the five-factor model and its applications. *J. Pers.* 60 175–215. 10.1111/j.1467-6494.1992.tb00970.x 1635039

[B40] Minnema L. (2014). Correlations between types of culture, styles of communication and forms of interreligious dialogue. *HTS Teol. Stud.* 70:2604 10.4102/hts.v70i1.2604

[B41] Muck P. M. (2003). *Der Interpersonale Circumplex als Grundlage einer Eigenschaftstheorie der Interpersonalität im Beruflichen Kontext [The Interpersonal Circumplex As A Basis For A Trait Theory Of The Interpersonal Domain For The Occupational Context].* Berlin: Springer.

[B42] Mulaik S. A. James R. L. Van Alstine J. Bennett N. Lind S. Stilwell C. D. (1989). Evaluation of goodness-of-fit indices for structural equation models. *Psychol. Bull.* 105 430–445.

[B43] Norton R. (1983). *Communicator Style: Theory, Applications and Measures.* Beverly Hills: Sage.

[B44] Norton R. W. (1978). Foundation of a communicator style construct. *Hum. Commun. Res.* 4 99–112. 10.1111/j.1468-2958.1978.tb00600.x

[B45] Ntoumanis N. Thøgersen-Ntoumani C. Quested E. Hancox J. (2017). The effects of training group exercise class instructors to adopt a motivationally adaptive communication style. *Scand. J. Med. Sci. Sports* 27 1026–1034. 27283879 10.1111/sms.12713

[B46] Ong L. M. de Haes J. C. Hoos A. M. Lammes F. B. (1995). Doctor-patient communication: a review of the literature. *Soc. Sci. Med.* 40 903–918. 7792630 10.1016/0277-9536(94)00155-m

[B47] Ramachandiran C. Mahmud M. (2019). Theorizing communicative styles on social media: an etymological shift. *J. Phys. Conf. Ser.* 1228:12073 10.1088/1742-6596/1228/1/012073

[B48] Riggio R. E. Carney D. R. (2003). *Social Skills Inventory.* Mountain View, CA: Mind Garden.

[B49] Rubin R. Martin M. (1994). Development of a measure of interpersonal competence. *Commun. Res. Rep.* 11 33–44. 10.1080/08824099409359938

[B50] Schermelleh-Engel K. Moosbrugger H. Müller H. (2003). Evaluating the fit of structural equation models: tests of significance and descriptive goodness-of-fit measures. *Methods Psychol. Res.* 8 23–74.

[B51] Schulz von Thun F. (2003). *Miteinander Reden.* Reinbek: Rowolth.

[B52] Sorenson R. L. Savage G. T. (1989). Signaling participation through relational communication: a test of the leader interpersonal influence model. *Group Organ. Manag.* 14 325–354.

[B53] Tomášková E. (2018). Expertise, leadership style and communication in interfunctional coordination. *Period. Polytech. Soc. Manag. Sci.* 26:11692 10.3311/PPso.11692

[B54] Trant A. A. Szekely B. Mougalian S. S. (2019). The impact of communication style on patient satisfaction. *Breast Cancer Res. Treat.* 176 349–356. 31025271 10.1007/s10549-019-05232-w

[B55] Waldherr A. Muck P. (2011). Towards an integrative approach to communication styles: the interpersonal circumplex and the Five-Factor theory of personality as frames of reference. *Communications* 36 1–27. 10.1515/COMM.2011.001

[B56] Wiggins J. S. (1979). A psychological taxonomy of trait-descriptive terms: the interpersonal domain. *J. Pers. Soc. Psychol.* 37 395–412.

[B57] Wiggins J. S. Phillips N. Trapnell P. (1989). Circular reasoning about interpersonal behavior: evidence concerning some untested assumptions underlying diagnostic classification. *J. Pers. Soc. Psychol.* 56 296–305.

[B58] Wiggins J. S. Trapnell P. Phillips N. (1988). Psychometric and geometric characteristics of the revised interpersonal adjective scales. *Multiv. Behav. Res.* 23 517–530. 10.1207/s15327906mbr2304_8 26761163

[B59] Yang Y. Kuria G. N. Gu D. X. (2020). Mediating role of trust between leader communication style and subordinate’s work outcomes in project teams. *Eng. Manag. J.* 1–14. 10.1080/10429247.2020.1733380

